# Death of M. Eugene Shaw, M. D.

**Published:** 1867-11

**Authors:** 


### Death of M. Eugene Shaw, M. D.

We are pained to announce the sudden death of Dr. M. Eugene Shaw, of Buffalo, who, but a few weeks since, left his practice in this city and joined the U. S. Army in California. On the 17th of October the stage coach in which he was riding, was attacked by a party of fifteen Indians, and Dr. Shaw was shot through the lungs, the wound soon proving fatal. The party were pursued near ten miles. but finally made good their escape, taking with them the body of Dr. Shaw, which they buried at Soda Lake.

Dr. Shaw was a young man in the prime and vigor of life, graduated from the medical department of the University of Buffalo in 1864, and was 26 years old at the time of his death. He was the only remaining son of Dr. Merrill H. Shaw of Buffalo, who, with his family and a wide circle of friends, are deeply bereaved by his sudden and violent death.

Dr. Shaw gave promise of great usefulness in his profession, and was already distinguished as a young physician for his attainments. In the war of the Rebellion he was so impatient for opportunity to share his part, that against the wishes of his friends he enlisted as a private. He was, however, soon detached from his Company and served as Acting Assistant Surgeon in the 89th N. Y. He afterwards served as Assistant Surgeon in the 116th N. Y., until the close of the war, when he commenced in this city the practice of his profession.

